# Intraoral Soft Tissue Lesions in 6-Year-Old Schoolchildren in Regions of Southern Ecuador: An Epidemiological Study

**DOI:** 10.3390/children11040406

**Published:** 2024-03-29

**Authors:** Eleonor Vélez-León, Alberto Albaladejo, Emilia Guerrero, Ginger Galván, María Melo

**Affiliations:** 1 Unidad Académica de Salud y Bienestar, Carrera de Odontología, Universidad Católica Cuenca, Cuenca 010107, Ecuador; mguerreroc@ucacue.edu.ec (E.G.); ginger.galvan@est.ucacue.edu.ec (G.G.); 2 Department of Surgery, Faculty of Medicine, University of Salamanca, 37007 Salamanca, Spain; albertoalbaladejo@usal.es; 3 Faculty of Medicine and Dentistry, Department of Stomatology, University of Valencia, 46010 Valencia, Spain; mapimeal@uv.es

**Keywords:** child, epidemiology, oral mucosa, prevalence, rural

## Abstract

Oral Mucosal Lesions (OMLs) are conditions of the oral mucosa that cause alterations in their presentation and pain in the affected patient, highlighting their importance for study. The aim of this research is to determine the prevalence of oral lesions in the Ecuadorian Austro. Descriptive statistics were used to associate variables, yielding statistically significant findings based on oral lesions, sex, and geographical environment. This study was conducted under the appropriate bioethical permissions. The results obtained correspond to the prevalence of lesions by province. Morona Santiago displays a rate of 17% for abscesses, surpassing the provinces of Azuay (13.50%) and Cañar (10.67%). However, gingivitis for pathology, Azuay stands out with 13.17%, while Cañar and Morona Santiago present 10.50% and 8.33%, respectively. There is also a predominant occurrence of abscesses in rural areas (41.17%) compared to urban ones (26.33%). Regarding sex, girls show a higher percentage of abscesses (41.17%) compared to boys who have a clear tendency to present GUM lesions (22.33%). The data indicates that in the studied provinces, geographical environment and sex are key variables to understand the distribution of oral lesions. These findings encourage us to continue pursuing this type of research, which contributes to improving the quality of life for children.

## 1. Introduction

Oral Mucosal Lesions (OMLs) encompass alterations in the oral mucosa, presenting as changes in color, surface abnormalities, inflammation, or loss of mucosal integrity [1,2]. The etiopathogenesis of these lesions ranges from infectious causes such as bacteria, fungi, and viruses to manifestations of systemic diseases, adverse drug effects, allergies, nutritional deficiencies, and chemical and mechanical lesions [1,3]. While most OMLs are benign, it is crucial to pay attention to those with malignant potential.

Despite affecting individuals of all ages and regions, there is a notable predisposition for children around the age of five to experience OMLs. This susceptibility during the preschool stage is attributed to psychological stress and anxiety caused by caregiver separation and exposure to new environments [4]. Additional risk factors include socioeconomic status, linked to nutritional status, increased stress, and lack of access to healthcare systems [5].

Consequently, attention to the pediatric population with OMLs is crucial, as children experience painful symptoms, impairment in physiological functions such as chewing, swallowing, and phonation, as well as aesthetic and psychological issues. These impacts translate into a deterioration in the quality of life, with a particular decrease in academic performance [1,2,4].

Although OMLs represent the third common pathology after caries and periodontal disease [6], scientific evidence regarding their prevalence is limited and controversial, especially in children and in Latin America. Globally, the prevalence of these lesions ranges from 5% to 65%, differences associated with methodological diversity among studies and sociodemographic characteristics of each region [2]. A review of global prevalences of OMLs, based on clinical studies, suggests that common oral lesions in children include aphthous ulcers (1.82%), traumatic ulcers (1.33%), and herpetic ulcers (1.33%), with the majority of reviewed studies from Europe and America [3] In Brazil, a study in pediatric patients reports a prevalence of 24.9% of oral lesions in children under 4 years old and 30.3% in children aged 5 to 12 years, with geographic tongue being the most common lesion, followed by traumatic bite lesions [7]. In Chile, a prevalence of 37.6% of OMLs is observed in children aged 3 to 13 years, with minor aphthae being the most common lesions [8] Regarding the location of OMLs, investigations in the United States reveal that the lip is the most frequent site of occurrence (30.7%), followed by the dorsum of the tongue and the oral mucosa at 14.7% and 13.6%, respectively [9] The lack of standardization in methodology and diagnostic criteria in original and review studies makes it challenging to define the true prevalence of OMLs in pediatric patients.

It is imperative to highlight that dental professionals must receive adequate training to diagnose OMLs, aiming to provide timely treatments. Additionally, delving into research on the prevalence of OMLs in pediatric populations is crucial to accurately understand their etiology and characteristics. This understanding will facilitate the improvement or establishment of protocols for oral health prevention and early intervention, as recommended by the World Health Organization [10] Considering the association between socioeconomic status and OML prevalence [4,11] the purpose of this research is to determine the prevalence in children from urban and rural environments in the provinces of Ecuador, marking the first study attempting to quantify these lesions in the country.

The central hypothesis of this study posits that the geographical environment and gender exert a significant influence on the prevalence and diversity of oral mucosal lesions (OMLs) observed in 6-year-old children. It is postulated that these significant differences are not random, but are directly linked to the aforementioned variables.

## 2. Materials and Methods

This descriptive, observational, and cross-sectional study is part of an epidemiological investigation conducted in 2019 with children from the southern region of Ecuador. The execution of this research adhered to the ethical principles outlined in the Helsinki Declaration and data protection standards.

It is important to note that approval was obtained from the Ethics Council of the Catholic University of Cuenca under its respective code No. 048 C.D-2019. Approval date: 14 February 2019. Before the study commenced, clear information was provided to the legal guardians of the children regarding the nature, objectives, and procedures of the research. Consequently, parents or guardians provided their informed consent, an essential requirement for participation in the study.

The dependent variables calculated for the study were the total prevalence of oral lesions in soft tissues. This would be the main dependent variable representing the presence or absence of oral lesions in soft tissues in the population of 6-year-old children.

Prevalence of Specific Types of Lesions (Codes 1 to 7) were secondary dependent variables representing the presence or absence of specific types of lesions. For this study, additional codes were adapted to complement those specified by the WHO [10]. These supplementary codes were based on criteria allowing identification in the field through visual examination.

Code 1: Ulcers

Code 2: Gingivitis

Code 3: Lip trauma

Code 4: Geographic tongue

Code 5: Cheilitis

Code 6: Pigmented lesion

Code 7: Other disorders

### 2.1. Sample

To carry out this research, a multi-stage stratified sampling method was employed to ensure fair and representative selection of the target population. This study is part of a broader project with a total sample of 1938 children; however, for this specific study focused on 6-year-old children, a sample of 600 students was used, with 297 children from urban areas and 303 children from rural environments attending public schools in three provinces within the southern region of Ecuador. This allowed for a confidence level of 95% with a margin of error of 0.05%.

### 2.2. Criteria for Selection

Participants in this study underwent a meticulous selection process, following strict criteria to ensure a well-defined cohort. Inclusion criteria stipulated that individuals must be precisely 6 years old, regardless sex, and originate from the provinces of Cañar, Morona Santiago, and Azuay, encompassing both rural and urban contexts. Rigorous scrutiny was employed to confirm compliance with these inclusion criteria, simultaneously excluding any participant with legal or systemic impediments, thus maintaining the principles of informed consent.

It is imperative to highlight that selected children were specifically chosen for their absence of systemic diseases. This stringent criterion was applied to maintain the homogeneity of the study group and ensure that the collected data reflected the target age group without confounding factors related to systemic health. Consequently, a meticulously curated dataset with 600 observations was assembled, representing the diverse landscapes of both rural and urban environments within the provinces under investigation.

### 2.3. Calibration

The calibration process was conducted with six specialized dentists who were calibrated following the criteria of the World Health Organization (WHO) for assessing soft tissue lesions of the oral cavity. Additionally, in this study, specific codes were adapted to complement those given by the WHO. These additional codes were based on criteria allowing field identification through visual examination exclusively. During the clinical phase, each specialist examined two groups of children, assisted by a previously trained dental student for information collection.

Different anatomical areas belonging to the oral mucosa were examined using the following codes and criteria specific to the calibration of Oral Mucosal Lesions (OMLs):

Code 1: Ulcers—What is the size of the ulcer? Is it increasing or decreasing? Does it have a particular shape? Are the edges defined or irregular? Does the patient experience pain? What is its intensity? Where is the ulcer located?

Code 2: Gingivitis—Is there inflammation, redness, or bleeding in the gums? Is there a buildup of bacterial plaque along the gum line?

Code 3: Lip Trauma—Are there visible signs of wounds, bruises, or injuries on the lips? Has the patient indicated the cause of the trauma?

Code 4: Geographic Tongue—Are there smooth areas with raised edges and depressed centers on the tongue? Does the patient report pain or other discomfort? Have there been changes in the pattern or appearance of the tongue over time?

Code 5: Cheilitis—Are there symptoms such as swelling, redness, or fissures in the lips? Does the patient have dry or cracked lips? Has any medical condition or habit contributing to cheilitis been identified?

Code 6: Pigmented Lesion—What is the color of the lesion? Has its color changed over time? What is the size and shape of the lesion? Have there been any changes in the lesion since its appearance?

Code 7: Other Disorders

### 2.4. Examination

Before the clinical examination, participants’ data were recorded on pre-prepared forms, including general information such as full name, sex, geographical location, and type of locality (urban-rural). The examination took place in spaces within educational institutions, under standardized conditions recommended by the World Health Organization (WHO). Due to the absence of dental equipment, the examination was conducted in a chair with a straight back, with the operator facing the child. The following items were used in each examination: a flat intraoral mirror No. 5, a pair of nitrile gloves, disposable surgical masks for each patient, a headlamp, gauze, and cotton. Calibrated examiners conducted the examinations while assistants completed the data collection form.

### 2.5. Diagnosis of OMLs

During the diagnosis process of oral mucosal lesions (OMLs), the criteria established by the World Health Organization (WHO) were used, along with additional criteria, following a systematic order. The assessment began with the inspection of the mucosa and lip grooves, covering the inside of the corners of the mouth and the oral mucosa. Additionally, a detailed examination of the tongue and the floor of the mouth was conducted, and finally, the hard and soft palate were assessed.

### 2.6. Statistical Analysis

Data collection was managed using Microsoft Excel 2019, where descriptive statistics, including absolute and relative frequencies of the analyzed variables, were calculated. To assess the association between oral lesions and factors such as sex, living environment, and geographical location, the chi-square test was employed. We used RStudio software® (version 4.3.0) to generate visual graphs illustrating these findings. Chi-square tests were conducted to assess independence between various categorical variables, such as province, environment, and sex in relation to the prevalence of lesions.

- Chi-square for Province and Lesion: X^2^ = 1.7147 × 10^2^df = 10 *p* < 2.2 × 10^(-16)
- Chi-square for Environment and Lesion: X^2^ = 4.3691 × 10^2^df = 5 *p* < 2.2 × 10^(-16)
- Chi-square for Sex and Lesion: X^2^ = 4.0155 × 10^2^df = 5 *p* < 2.2 × 10^(-16)

Two hypotheses were posited: the Null Hypothesis (H0), which posits that the geographical environment and gender do not significantly influence the prevalence and diversity of OMLs, and the Alternative Hypothesis (H1), suggesting a significant influence of these variables on the prevalence and diversity of OMLs.

## 3. Results

### 3.1. Distribution of the Participants

A study was conducted with 600 schoolchildren, 6 years of age, from urban and rural areas of the provinces of Cañar, Morona Santiago, and Azuay. In selecting the participants, equitable stratification was used to ensure a balanced representation of both environments (Table 1).

### 3.2. Prevalence of OMLs in the Oral Cavity in the Study Population

**Figure 1:**
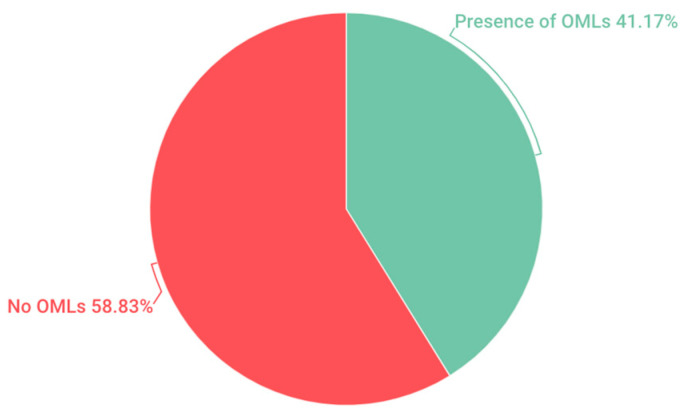
Total OMLs.

In a general context, within the study population, it was observed that 41.17% of the analyzed schoolchildren exhibited some form of oral soft tissue lesions, while the remaining 58.83% showed no alterations (Figure 1).

### 3.3. Prevalence of OMLs at the General Level

**Figure 2:**
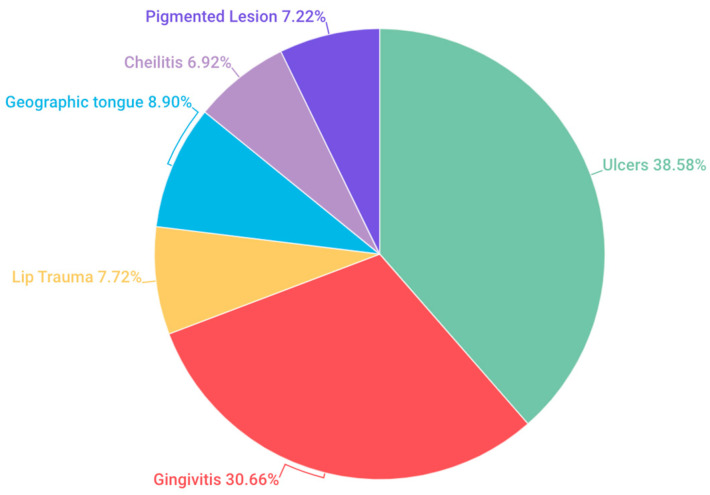
Prevalence of OMLs at the general level.

La current research revealed overall prevalence rates, highlighting 38.58% for ulcers, followed by 30.66%for gingivitis, 7.72% for lip trauma, 8.9% for geographic tongue, along with 6.92% for cheilitis, and finally, 7.22% for pigmented lesions (Figure 2).

### 3.4. Prevalence Acorde to Province

**Figure 3:**
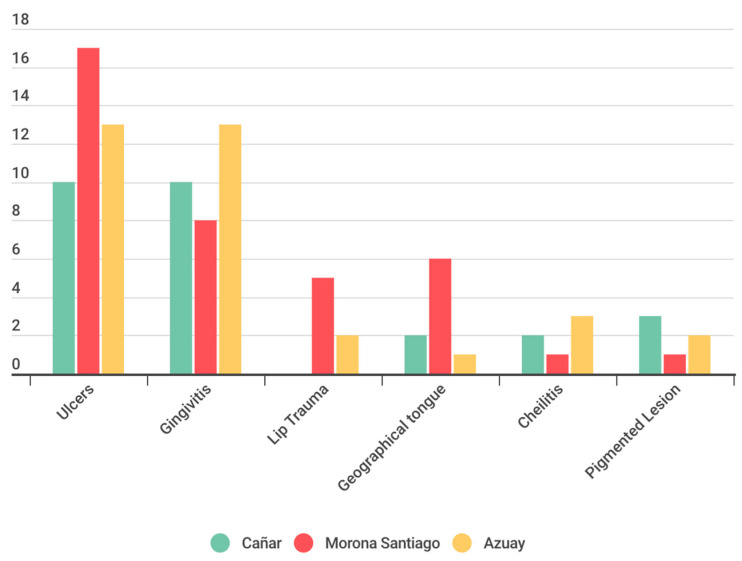
OMLs experience according to province.

The results indicate that geographical location, in this case, the province, plays a statistically significant role in the prevalence of different types of lesions in the studied population. There is notable variability among provinces regarding the prevalence of these lesions. Specifically, ulcers are most prevalent in Morona Santiago, at 17%, closely followed by Azuay at 13.50%, and Cañar at 10.67%. On the other hand, gingivitis lesions are more prevalent in Azuay, at 13.17%, followed by Cañar at 10.50%, and Morona Santiago at 8.33%. The results obtained in this study indicate that there is sufficient statistical evidence to reject the null hypothesis (H0), which posited the absence of a significant relationship between the province of origin and the type of lesion investigated. These findings are supported by statistical analysis, which yields a chi-squared value of 171.47 with 10 degrees of freedom and a *p*-value significantly less than 0.05 (Figure 3).

### 3.5. Prevalence of OMLs Based on Environmental Setting

**Figure 4:**
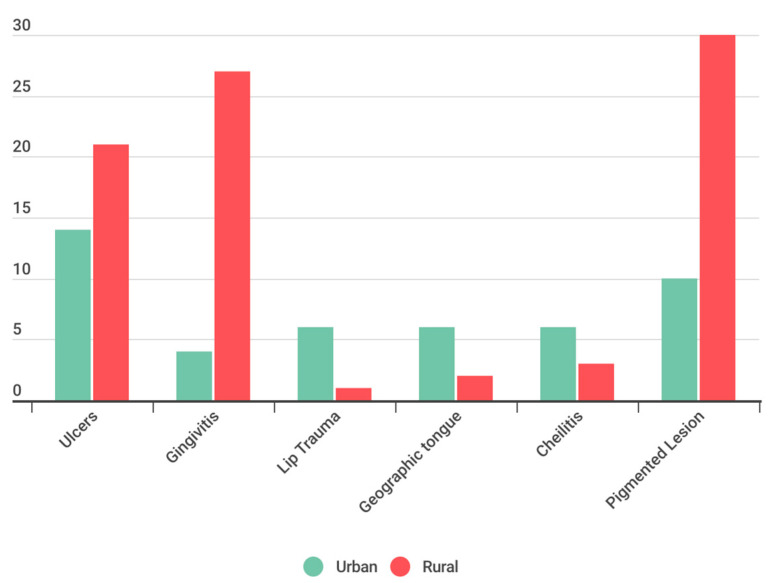
OMLs based on environmental setting.

In this analysis, it is evidenced the presence of a statistically significant relationship between the residential environment and the type of experienced lesions, (The Chi-squared value is 436.91 with 5 degrees of freedom. The *p*-value is significantly less than 0.05 (actually, less than 2.2 × 10^(-16)), suggesting that we can reject the null hypothesis 0H0, which posited that there was no significant relationship between the setting (urban or rural) and the type of lesion. Leading to the rejection of the null hypothesis. Specifically, it is observed that ulcers lesions are more common in rural settings (27.17% of cases), while gingivitis lesions predominate in rural areas (27.33% of cases) (Figure 4).

### 3.6. Experience of OMLs Based on Sex

**Figure 5:**
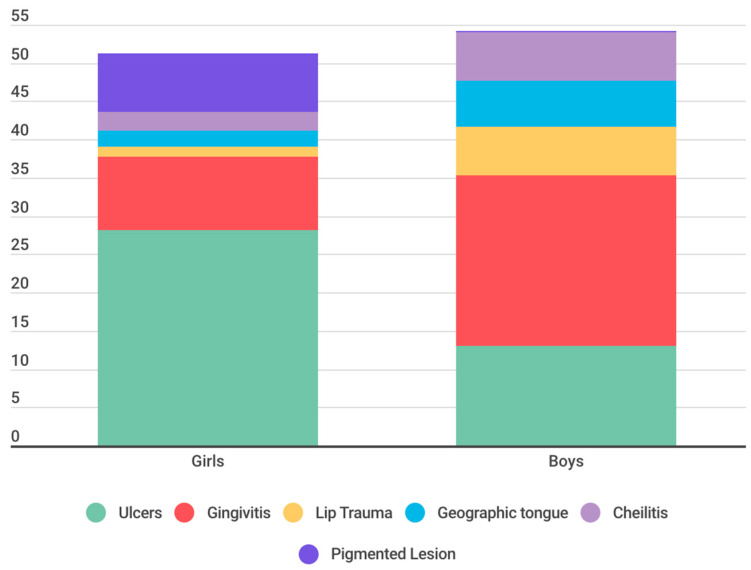
Experience if OMLs according to sex.

The prevalence of lesions shows differences between boys and girls. For example, girls have a prevalence of 28.17% for ulcers, while boys show a prevalence of 22.33% for gingivitis lesions. In terms of statistical analysis, the chi-square value is 401.55 with 5 degrees of freedom. The *p*-value is significantly less than 0.05, specifically less than 2.2 × 10^(-16), leading us to reject the null hypothesis (H0) that there was no significant relationship between sex and type of lesion (Figure 5).

## 4. Discussion

This research highlights the prevalence of oral mucosal lesions in 6-year-old schoolchildren and their correlation with sociodemographic factors, emphasizing the importance of addressing this issue. Age emerges as a critical factor in the analysis of oral mucosal lesions due to its direct influence on susceptibility, manifestation, and progression of these conditions. In pediatric populations, age can reflect different levels of exposure to risk factors, variability in immune response, and disparities in oral hygiene habits. These variations are essential for understanding the sociodemographic epidemiology of oral lesions and designing preventive interventions [6,12,13,14].

The significance of the age of 6 as a turning point in the study of oral mucosal lesions lies in the fact that this period coincides with the commencement of formal education, implying significant changes in the sociodemographic environment and habits of children. This sociodemographic transition can increase exposure to pathogens and alter oral hygiene practices, impacting the incidence of oral lesions. Furthermore, the eruption of permanent teeth begins around this age, influencing susceptibility to certain sociodemographic oral health conditions [6,12,13,14].

In the pediatric population examined in this study, comprising 6-year-old children, a prevalence of 41.17% of OMLs was recorded. This result bears resemblance to previous research conducted in the pediatric population, such as the study conducted by Viera in Brazil (40.7%) [11] Yánez in Chile (37.6%) [8], as well as the studies by Crivelli and García [15,16] who reported incidences of 39.04% and 38.94%, respectively, in Argentina and Spain. However, a wide variation in the prevalence rates of OMLs is observed when compared to research conducted in the United States. Sullman reported a prevalence of 6.89% [9], while Kleinman recorded 4% [17]. Additionally, notable discrepancies are observed with Yao’s study in China, which presented a prevalence of 1.8% [4], and with the study in Mexico conducted by Espinoza, where a 7.4% [18] prevalence was found.

The overall prevalence of OMls shows considerable variation, ranging between 5% and 65% in various regions and countries worldwide [2]. The observed disparity in prevalence percentages, as mentioned earlier, can be attributed to a multitude of factors inherent to each region under study. The diverse sociodemographic characteristics of different regions, encompassing variations in cultural practices, economic status, and access to healthcare, play a crucial role in influencing the prevalence rates of OMLs. Population habits, which encompass oral hygiene practices, dietary choices, and lifestyle factors, further contribute to the divergent prevalence figures. Additionally, the wide range of prevalence rates is exacerbated by the varied methodologies employed in these studies. Differences in study design, sampling methods, and diagnostic criteria contribute to the observed variations. Moreover, the lack of standardization in clinical and diagnostic criteria is a significant contributing factor, leading to inconsistencies in the identification and classification of OMLs across diverse studies [1,7].

In our investigation, oral ulcers emerged as the most prevalent OMLs, reaching a prevalence of 38.58%. This finding aligns with the research of Yao (0.4%), [4] Kleinman (1.23%) [17] and Oliveira (29.3%) [19] where oral ulcers were also identified as the most frequent alteration. However, notable differences in percentages were observed among these studies. Conversely, no congruence was found with the comprehensive review conducted by Hong and colleagues, who provided data on commonly observed alterations in children from various regions worldwide [3]. In that study, ulcers ranked third in prevalence, ranging from 0.3% to 4.8%, following trauma-associated lesions (2.5–4.1%) and fissured tongue (0.3–4.0%), which were the most frequent.

From our perspective, the extensive array of potential causes for oral ulcers in children is intricately connected to the observed diversity in prevalence rates. Furthermore, the intermittent nature of these lesions adds complexity to the comparison of rates. Additionally, given that our study was purely observational, we were limited to a clinical diagnosis of the ulcers, without delving into their diverse origins. We deem it essential for future research to conduct complementary longitudinal studies to determine the etiology of the observed ulcers. Discrepancies in the sampling frame, diagnostic criteria, training, calibration of personnel, and examination features also significantly contribute to the variations observed in results across different studies.

The studies by Záror Sánchez and Olga Taboada Aranza identified key factors related to the prevalence of gingivitis in children and adolescents [20]. In Záror Sánchez’s study, a high prevalence of 93% of gingivitis was found, suggesting the significant influence of oral hygiene and the presence of plaque and caries in the development of this condition. On the other hand, Olga Taboada Aranza’s work, with a prevalence of 39% of gingivitis, emphasizes the importance of considering additional factors such as the subjects’ age and possibly other aspects related to oral hygiene habits and overall health.

Comparing these findings with our research, where a prevalence of 30.66% of gingivitis was identified, suggests the need for more detailed studies to understand how these specific factors may influence the variability in the prevalence of gingivitis in different age groups.

Our study revealed a prevalence of 7.72% for lip lesions. When comparing this result with Hua-Qiu Guo’s study [21], which found a prevalence of 60.1% in a population of infants and schoolchildren, a significant disparity in the reported percentages is evident. This suggests that lip lesions can vary significantly among different age groups and studied populations.

Regarding traumatic lesions, Alessandra Majorana found a prevalence of 17.8% in a similar population, a figure surpassing that reported in our study [22]. On the other hand, Besa’s study found prevalences of 2.01% in children aged 0 to 4 years and 2.58% in children aged 5 to 9 years, contrasting with our research findings but indicating a trend toward lower incidence in younger age groups.

These comparisons underscore the importance of considering differences in the prevalence of oral lesions between studies and populations, influenced by demographic, geographic, cultural, and etiological factors, such as habits like cheek biting, morsicatio buccarum, tongue or lip sucking, biting objects (such as pens, toys), or local injuries (orthodontic devices, sharp fillings, fractured teeth). It is crucial to analyze these results collectively to gain a better understanding of the epidemiology and variability of oral lesions across different population groups.

Our study revealed a prevalence of 8.9% for geographic tongue in the studied population. When comparing these results with Alessandra Majorana’s study [22], which reported a prevalence of 11.89% in a population aged 0 to 12 years, a difference in percentages is observed, but with a relatively similar incidence between both investigations. Furthermore, contrasting our finding with Bessa’s study [7] which found a prevalence of 30.5%, significant variation in the reported data is evident. This suggests that the prevalence of geographic tongue can vary notably between different age groups or geographical regions studied.

On the other hand, Riobo’s study showed a variability of results ranging from 0.6% to 21% in the pediatric population, reflecting the diverse incidences of geographic tongue in different populations and contexts [23].

These comparisons underscore the importance of considering variability in the prevalence percentages of geographic tongue in different studies and populations, which may be influenced by diverse factors such as age, geographical location, oral health habits, and the methodology used in the research. Analyzing these disparities is crucial for gaining a more comprehensive understanding of this condition and its distribution across different populations.

When comparing our prevalence result of cheilitis (6.92%) with the study by Linares-Vieyra C et al., which reported a prevalence of 41.1%, a marked difference in the prevalence percentages of cheilitis between the two studies is evident [24]. While our study found a relatively low prevalence of cheilitis, the study by Linares-Vieyra C et al. showed a significantly higher incidence in the studied population. This disparity in the results suggests that cheilitis may occur more frequently in the population analyzed in the study by Linares-Vieyra C et al. compared to the population in our study. These differences in findings highlight the importance of considering variability in the incidence of cheilitis in different populations and the need to further investigate the underlying factors that may influence the prevalence of this condition in different contexts.

Regarding pigmented lesions, the intensity of melanin pigmentation varies depending on the amount of pigment and its depth, ranging from brown (superficial location) to black or blue (deep location). Oral pigmented lesions can originate from an abnormal accumulation of pigments usually present in the oral mucosa (melanin) or foreign to it (exogenous and endogenous pigments). When comparing our results on the prevalence of pigmented lesions (7.22%) with the findings of Linares-Vieyra C. et al. (8.47%), a relatively small difference in the reported figures is observed [24].

In the analysis of the distribution of anomalies in the oral mucosa among the three provinces under study, similarities were identified; however, a significantly higher prevalence of ulcers in Morona Santiago and a greater prevalence of gingivitis in Azuay were notable. Additionally, a statistically significant disparity was observed when contrasting the prevalence OMLs between urban and rural environments. In this regard, ulcers and gingivitis were identified more frequently in the rural setting of the three examined provinces. The obtained results suggest a statistically significant relationship between the sociodemographic and socioeconomic characteristics of the population and the prevalence of OMLs. This finding supports the conclusions of Viera [7], although it differs from the results obtained by Yao [4], where no relationship was found between these factors and the prevalence of OMLs.

In the study on the prevalence of oral mucosal lesions (OMLs) in 6-year-old children in Ecuador, valuable insights were uncovered that significantly contribute to understanding the issue within specific socio-demographic and geographical contexts. The benefits derived from this research include the identification of a high prevalence of oral ulcers as the most common manifestation of OMLs in the studied population, and the elucidation of the influence of rural environments on the incidence of these lesions. This knowledge is crucial for developing specific prevention and care strategies that can improve the oral and overall health of this vulnerable population.

However, the study is not without limitations that need to be addressed in future research to enrich the understanding and management of OMLs. These include:

1. Sample Representativeness: Although multi-stage stratified sampling aimed for representativeness, future studies could benefit from larger and more diversified samples, including a wider range of geographical regions to better capture the heterogeneity of the Ecuadorian pediatric population.
2. Depth in Environmental Classification: The distinction between rural and urban environments could be more nuanced, incorporating detailed analyses of how specific socio-economic factors and living conditions influence the prevalence and types of OMLs.
3. Etiological Understanding: The study was limited to observing prevalence without delving into the specific etiology of OMLs. Future research should explore underlying causes and associated risk factors through longitudinal study designs or case-control studies.
4. Standardization in Diagnosis: Variability in diagnostic criteria and examiner calibration may affect the consistency of results. Adopting standardized protocols and ensuring the training and periodic recalibration of examiners are crucial steps to improve the reliability of diagnoses.

Addressing these limitations through rigorous methodological strategies and a more detailed focus on etiology and environment classification will significantly contribute to the field of pediatric oral health. Not only will this allow for a better understanding of OMLs and their determinants, but it will also facilitate the implementation of more effective interventions to prevent and treat these conditions in children, thereby improving their quality of life and overall well-being.

## 5. Conclusions

All tests resulted in a *p*-value significantly less than 0.05, leading to the rejection of the null hypothesis H0 and indicating significant differences in lesion prevalence based on province, environment, and gender. The study’s findings reveal a significantly. High prevalence of Oral Mucosal Lesions (OML) in the studied Ecuadorian pediatric population, with ulcers being the most frequently observed manifestation in this group. Furthermore, a statistically significant relationship was identified between the prevalence of OMLs and the rural environment, indicating an increased incidence of these lesions in rural communities compared to urban areas.

## Figures and Tables

**Table 1:** Distribution of the Participants.

	Cañar	Morona Santiago	Azuay	Total
Urban	100	99	98	297
Rural	100	102	101	303
Total	200	201	199	600

## Data Availability

Base de datos https://docs.google.com/spreadsheets/d/1NvyztkqtcBPzn6eljLwnUJnlrY-ezYRZvlVlBPAJC3I/edit#gid=1731804063 (accessed on 1 February 2024).
